# Incidence trends of oral cavity, oropharyngeal, hypopharyngeal and laryngeal cancers among males in Taiwan, 1980–2019: a population-based cancer registry study

**DOI:** 10.1186/s12885-023-10651-6

**Published:** 2023-03-06

**Authors:** Yu-Shiun Tsai, Yong-Chen Chen, Tzu-I Chen, Yu-Kwang Lee, Chun-Ju Chiang, San-Lin You, Wan-Lun Hsu, Li-Jen Liao

**Affiliations:** 1 School of Medicine, College of Medicine, Fu-Jen Catholic University, New Taipei City, 242 Taiwan; 2 Master Program of Big Data Analysis in Biomedicine, College of Medicine, Fu Jen Catholic University, New Taipei City, 242 Taiwan; 3 Data Science Center, College of Medicine, Fu Jen Catholic University, New Taipei City, 242 Taiwan; 4 Graduate Institute of Business Administration, College of Management, Fu Jen Catholic University, New Taipei City, 242062 Taiwan; 5 Division of General Surgery, Department of Surgery, National Taiwan University Hospital, Taipei, 100 Taiwan; 6 Graduate Institute of Epidemiology and Preventive Medicine, College of Public Health, National Taiwan University, Taipei, 10055 Taiwan; 7 Otolaryngology Head and Neck Surgery, Far Eastern Memorial Hospital, No. 21, Sec. 2, Nanya S. Rd., Banciao Dist., New Taipei City, 220 Taiwan; 8 Head and Neck Cancer Surveillance & Research Group, Far Eastern Memorial Hospital, New Taipei, Taiwan; 9 Department of Electrical Engineering, Yuan Ze University, Taoyuan, Taiwan

**Keywords:** Head and neck cancers, Incidence, Secular trend, Age-period-cohort effect

## Abstract

In a country with a high prevalence of cigarette smoking, betel chewing, and alcohol drinking, cancers of the oral cavity, nasopharynx, and larynx were the fourth, twelfth and seventeenth leading causes of cancer death, respectively, for men in 2020. We analyzed patients with head and neck cancer from 1980 to 2019 from the Taiwan Cancer Registration Database and discussed the annual average percent change, average percent change, age period, and birth cohort. Obvious period effects and birth effects are seen in oral, oropharyngeal, and hypopharyngeal cancer; however, the most significant period effect was seen between 1990 and 2009, which mainly reflects the consumption of betel nuts per capita. In addition, the period effect lessens after 2010 in oral cancer and hypopharyngeal cancers, while oropharyngeal cancers remain an obvious period effect, which results from the rising prevalence of HPV. Due to the high prevalence rate of betel quid chewing and cigarette smoking in the 1990s, the government executed several acts. As a result, the age-adjusted incidence rates of oral, oropharyngeal, and hypopharyngeal cancers have flattened since 2010, which can be explained by the declining cigarette smoking rate. The strict policy indeed shows an obvious effect on the head and neck cancer incidence rates, and we expect to see a further decline in the future.

## Introduction

Head and neck cancers are one of the most common types of cancer worldwide. According to GLOBOCAN 2020, head and neck cancers, including lip and oral, oropharyngeal, hypopharyngeal, and laryngeal cancer, accounted for a total of 744,994 new cases and 364,339 new cancer deaths in 2020 worldwide, which represents 9.6% of new cancer cases and 4.72% of new cancer deaths in all populations [1]. Specifically, they accounted for 14.6 and 4.39% of new cancer cases in men and women in 2020 worldwide, respectively; meanwhile, they accounted for 7.22 and 2.15% of new cancer deaths in men and women in 2020 worldwide, respectively [1]. In Taiwan, cancer of the oral cavity was the fourth leading cause of cancer death for men, while laryngeal cancer was the seventeenth leading cause of cancer death for men in 2020 [2].

Smoking, alcohol consumption, and betel quid chewing are three major risk factors for head and neck cancer [3–5]. A study showed that the odds ratios of smoking, alcohol consumption, and betel quid chewing attributed to head and neck cancer were 1.58, 8.23, and 2.29, respectively; in addition, individuals who had all 3 habits exhibited a 20.6 odds ratio of head and neck cancer that was higher than that of individuals who had 1 or 2 habits [5]. Other proven carcinogenic factors include human papillomavirus infection, poor oral hygiene, while higher intake of vegetables has a protective role [6, 7]. Due to the high prevalence of smoking, alcohol consumption, and betel quid chewing habits in Taiwan, the incidence of head and neck cancer has increased significantly in the past 40 years. A previous study performed by Wan-Lun Hsu showed that the age annual percent change in head and neck cancer incidence between 1980 and 2014 was 5.4% per year among males and 3.1% among females [8]. As a result, the Taiwanese government not only encourages farmers to replace areca trees with other crops but also enforces the Waste Disposal Act to forbid betel quid spitting. In addition, the Taiwanese government also promulgated the Tobacco Hazards Prevention Act in 1997 and elevated tobacco health and welfare surcharges several times. Moreover, a free oral cancer screening program for high-risk populations has been executed since 2004.

Previous studies in Taiwan have analyzed the trends in the incidence of head and neck cancers, but fewer studies have further discussed birth and period effects and the changes due to new policy-making [8]. This study aimed to investigate the interaction of three major risk factors resulting in cancer incidence change and further investigate the change of risk factors among different time periods, age groups, and birth cohorts. To achieve this goal, we not only used 40 years of high-quality data from the Taiwan cancer registration database but also provided a comprehensive and thorough investigation of the four major head and neck cancers including oral, oropharyngeal, hypopharyngeal, and laryngeal cancer. The annual average percent change, average percent change, and age-period-cohort effect are discussed to find the birth and period effects that impact these three major risk factors. In this study, we further investigated the birth and period effects of head and neck cancers from 1980 to 2019 and surveyed related head and neck cancer public health policies.

## Materials and methods

Head and neck cancer cases between 1980 and 2019 were obtained from the national Taiwan Cancer Registration (TCR) database (https://twcr.tw/). The Taiwan Cancer Registration (TCR) database has collected newly diagnosed cancer cases from hospitals with 50 or more beds in Taiwan since 1979 [9, 10]. In addition, the completeness of the TCR, the percentage of cases with death certificates, and the percentage of morphological verification in 2016 were respectively 98.4, 0.9, and 93%, while the completeness was measured by all registered cancer cases divided by all potential cancer cases from profiles of death certificate, NHI catastrophic illnesses, and four major cancer screening programs [9]. All cases in this analysis were classified based on the International Classification of Diseases for Oncology, third edition (ICD-O-03) [11]. Head and neck cancer cases were categorized into oral cancer (C00, C02, C03, C04, C050, C058, C059, and C06, excluding C024), oropharyngeal cancer (C01, C024, C051, C052, C09, C10, C142, and C148), hypopharyngeal cancer (C12, C13, and C140), and laryngeal cancer (C32).

Based on the 2000 World Health Organization standard population, the age-adjusted incidence rate in men from 1980 to 2019 was only analyzed due to the low incidence in women in Taiwan. For the analysis of long-term trends, the age-specific incidence rate from 1980 to 2019 was calculated for specific age groups, time periods, and birth cohorts. The age-specific incidence rate was classified into eighteen 5-year age groups (0–4, 5–9, 10–14, 15–19, 20–24, 25–29, 30–34, 35–39, 40–44, 45–49, 50–54, 55–59, 60–64, 65–69, 70–74, 75–79, 80–84, and 85+) and eight 5-year time periods (1980–1984, 1985–1989, 1990–1994, 1995–1999, 2000–2004, 2005–2009, 2010–2014, and 2015–2019). In addition, the birth cohort was divided into eleven birth groups (1930–1934, 1935–1939, 1940–1944, 1945–1949, 1950–1954, 1955–1959, 1960–1964, 1965–1969, 1970–1974, 1975–1979, and 1980–1984) and twelve 5-year age groups (30–34, 35–39, 40–44, 45–49, 50–54, 55–59, 60–64, 65–69, 70–74, 75–79, 80–84, and 85+). Moreover, to describe the linear change in the age-adjusted incidence rate from 1980 to 2019, a join point regression model was utilized to detect the change point and calculate the average annual percent change (AAPC) and annual percent change (APC) [10]. In addition, the 95% confidence intervals of the average annual percent change (AAPC) and annual percent change (APC) were analyzed. 95% confidence interval indicated 95% would fall between the upper limit and the lower limit, while 95% confidence interval including 0 showed statistically nonsignificant. The research protocol was approved by the Institutional Review Board of Fu-Jen Catholic University (No. C104014).

## Results

**Fig. 1:**
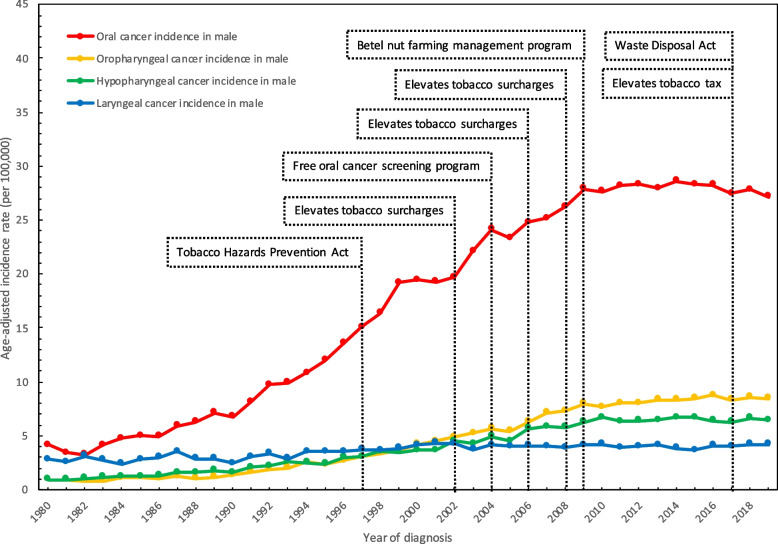
Long term trend of age-adjusted incidence rates of men in Taiwan during 1980 to 2019

We analyzed the age-adjusted incidence rates of oral, oropharyngeal, hypopharyngeal, and laryngeal cancer in men in Taiwan from 1980 to 2019. (Fig. 1) The incidence rate of oral cancer in men increased from 4.19 per 100,000 persons in 1980 to 27.19 per 100,000 persons in 2019, with an AAPC of 5.1% (95% CI = 4.4 to 5.8). Moreover, there was a significant increase from 1982 to 1999 and a steady increase from 1999 to 2009, with an APC of 10.1% (95% CI = 9.6 to 10.6) and of 4.5% (95% CI = 3.4 to 5.6), respectively (Fig. 1, Table 1).

**Table 1:** Joinpoint analysis of trends in the incidence rates for oral, oropharyngeal, hypopharyngeal, laryngeal cancer in males, Taiwan 1980–2019

Cancer types	Oral cancer	Oropharyngeal cancer	Hypopharyngeal cancer	Laryngeal cancer
AAPC (95% CI) 1980–2019	5.1% (4.4, 5.8)	5.7% (4.0, 7.5)	5.1% (4.7, 5.5)	1.2% (0.7, 1.6)
Trend 1, APC (95%)	1980–1982, − 6.2% (− 16.5, 5.4)	1980–1982, − 9.8% (− 21.3, 3.4)	1980–2008, 7.0% (6.7, 7.3) **	1980–2002, 2.1% (1.5, 2.7) **
Trend 2, APC (95%)	1982–1999, 10.1% (9.6, 10.6)**	1982–1985, 13.6% (− 0.9, 30.3)	2008–2019, 0.4% (−0.8, 1.5)	2002–2019, 0.0% (− 0.8, 0.8)
Trend 3, APC (95%)	1999–2009, 4.5% (3.4, 5.6)**	1985–1988, −1.2% (− 13.8, 13.2)		
Trend 4, APC (95%)	2009–2019, − 0.1% (− 1.0, 0.8)	1988–1999, 11.9% (10.7, 13.1)**		
Trend 5, APC (95%)		1999–2009, 7.3% (5.9, 8.6)**		
Trend 6, APC (95%)		2009–2019, 1.0% (0.0, 2.1)		

** indicates *p* < 0.001

In terms of oropharyngeal cancer, the incidence rate in men increased from 0.97 per 100,000 persons in 1980 to 8.46 per 100,000 persons in 2019, with an AAPC of 5.7% (95% CI = 4.0 to 7.5); in addition, there was a dramatic increase from 1988 to 1999 and from 1999 to 2009, with an APC of 11.9% (95% CI = 10.7 to 13.1) and 7.3% (95% CI = 5.9 to 8.6), respectively (Fig. 1, Table 1).

With regard to hypopharyngeal cancer, the incidence rate in men increased from 0.92 per 100,000 people in 1980 to 6.46 per 100,000 persons in 2019, with an AAPC of 5.1% (95% CI = 4.7 to 5.5). There was a significant upward trend from 1980 to 2008, with an APC of 7.0% (95% CI = 6.7 to 7.3) (Fig. 1, Table 1).

Regarding laryngeal cancer, the incidence rate in men increased from 2.8 per 100,000 persons in 1980 to 4.28 per 100,000 persons in 2002, with an APC of 2.1% (95% CI = 1.5 to 2.7). Then, the rate fluctuated to approximately 4 per 100,000 people from 2002 to 2019, with an APC of 0% (95% CI = − 0.8 to 0.8). Meanwhile, the total AAPC from 1980 to 2019 was 1.2% (95% CI = 0.7 to 1.6) (Fig. 1, Table 1).

**Fig. 2:**
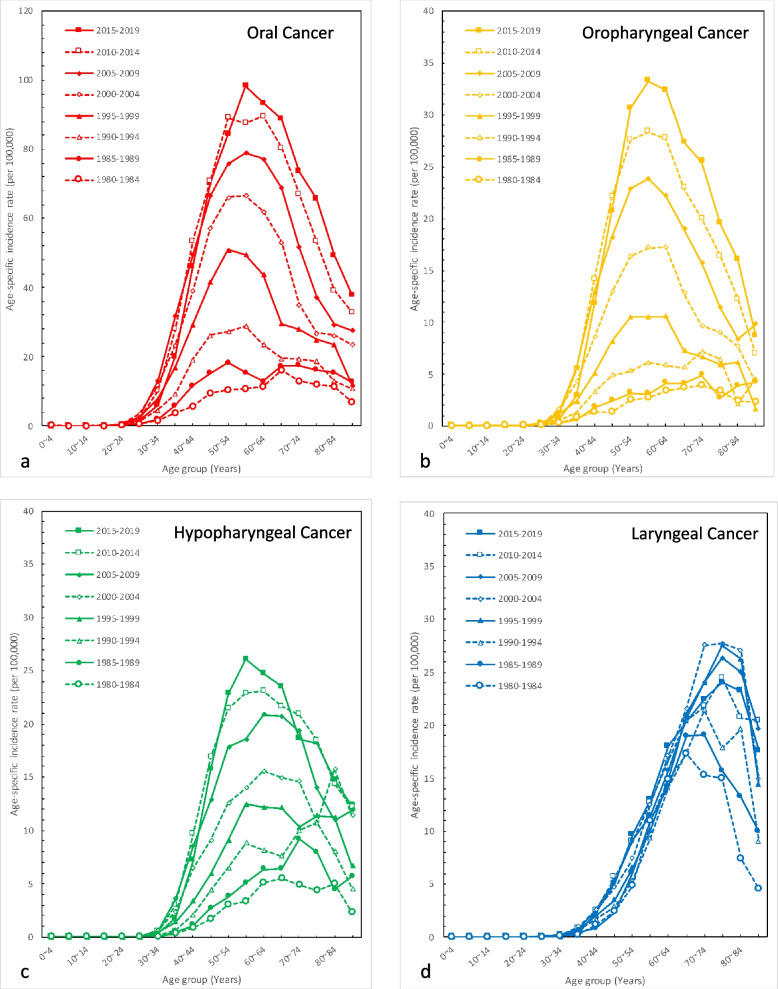
Age-specific incidence rates of men in Taiwan during 1980 to 2019. **a** Oral cancer; **b** Oropharyngeal cancer; **c** Hypopharyngeal cancer; **d** Laryngeal cancer

We investigated the age-specific incidence rates of oral, oropharyngeal, hypopharyngeal, and laryngeal cancer in men in Taiwan from 1980 to 2019. Concerning oral, oropharyngeal, and hypopharyngeal cancer (Fig. 2 a to c) in men, similar trends were observed: the more recent the period was, the higher the incidence rate, especially in the age groups of 45–50 to 70–74 years. Moreover, the most obvious period effect was observed from 1990 to 1995 to 2005–2009; in addition, after 2005–2009, the more recent the age period was, the less obvious the period effects were. Using the 60- to 64-year-old age group as an example, in 1980–1984, the incidence rates of oral, oropharyngeal, and hypopharyngeal cancer11.5, 3.3, and 5.1 per 100,000 persons, respectively. In 1985–1989, the incidence rates of oral, oropharyngeal, and hypopharyngeal cancer increased to 12.9, 4.1, and 6.3 per 100,000 persons, respectively. In 1990–1994, the incidence rates of oral, oropharyngeal, and hypopharyngeal cancer increased to 23.4, 5.9, and 8.1 per 100,000 persons, respectively. In 1995–1999, the incidence rates of oral, oropharyngeal, and hypopharyngeal cancer increased to 43.8, 10.6, and 12.2 per 100,000 persons, respectively. In 2000–2004, the incidence rates of oral, oropharyngeal, and hypopharyngeal cancer increased to 62.0, 17.2, and 15.5 per 100,000 persons. In 2005–2009, the incidence rates of oral, oropharyngeal, and hypopharyngeal cancer strongly increased to 77.3, 22.2, and 20.9 per 100,000 persons, respectively. In 2010–2014, the incidence rates of oral, oropharyngeal, and hypopharyngeal cancer increased more slowly to 89.7, 27.7, and 23.1 per 100,000 persons, respectively. In 2015–2019, the incidence rates of oral, oropharyngeal, and hypopharyngeal cancer steadily rose to 93.4, 32.3, and 24.7 per 100,000 persons, respectively.

In addition, the peaks in each period were observed earlier in recent periods. For example, the peak incidence rates of oral, oropharyngeal, and hypopharyngeal cancer in men occurred in 65–69 years (16.1 per 100,000 persons), 70–74 years (3.9 per 100,000 persons), and 65–69 years (5.5 per 100,000 persons) in the age group of 1980 to 1984; however, the peak incidence rates of oral, oropharyngeal, and hypopharyngeal cancer occurred in 55–59 years (98.2 per 100,000 persons), 55–59 years (33.3 per 100,000 persons), and 55–59 years (26.1 per 100,000 persons) in the age group of 2015 to 2019 (Fig. 2a to c).

For laryngeal cancer in men, earlier periods and recent periods do not exhibit an obvious trend. Among the same periods, younger age groups tended to have a lower incidence rate, especially the under 70–74 years group (Fig. 2d).

**Fig. 3:**
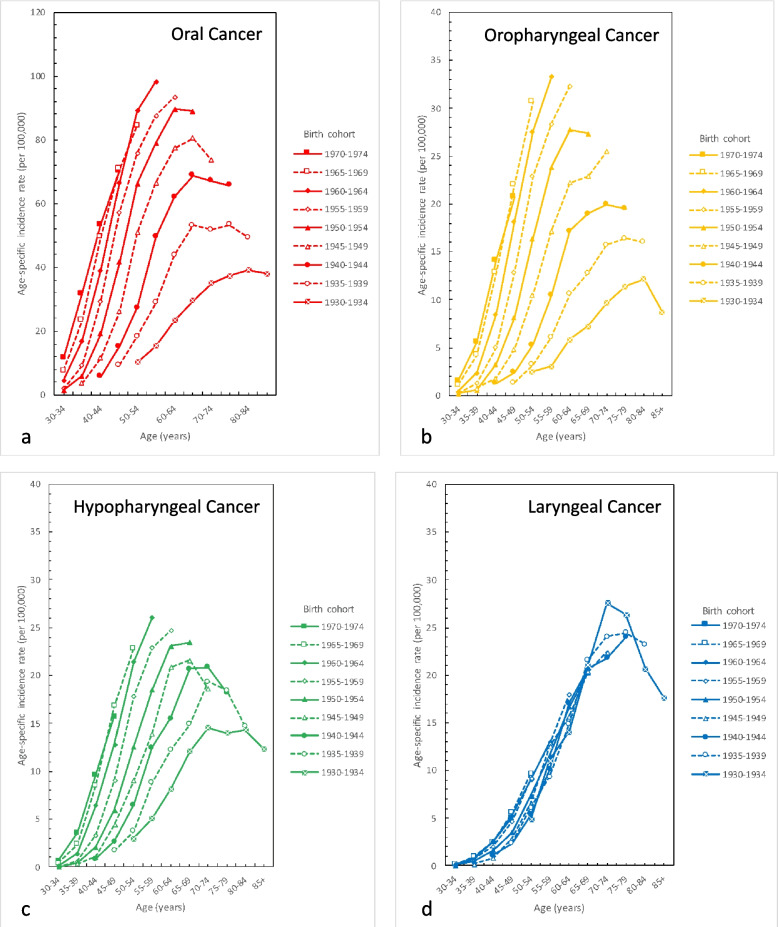
Age-specific incidence rates of men in Taiwan by birth cohort during 1980 to 2019. **a** Oral cancer; **b** Oropharyngeal cancer; **c** Hypopharyngeal cancer; **d** Laryngeal cancer

We surveyed the age-specific incidence rates of oral, oropharyngeal, hypopharyngeal and laryngeal cancer in men in Taiwan by birth cohort from 1980 to 2019 (Fig. 3a to d). The birth cohorts of oral, oropharyngeal, and hypopharyngeal cancer in men reveal that the older birth cohorts have a lower incidence rate than the younger cohorts (Fig. 3a to c). Using the 55–59 years age group as an example, in birth cohort 1930–1934, the incidence rates of oral, oropharyngeal, and hypopharyngeal cancer were 15.4, 3.1, and 5.1 per 100,000 persons, respectively. In birth cohort 1945–1949, the incidence rates of oral, oropharyngeal, and hypopharyngeal cancer were 66.6, 17.2, and 14.0 per 100,000 persons, respectively. In birth cohort 1960–1964, the incidence rates of oral, oropharyngeal, and hypopharyngeal cancer were 98.3, 33.3, and 26.1 per 100,000 persons, respectively.

In terms of laryngeal cancer, no apparent pattern was observed between the older and younger cohorts. Among the same cohort, a higher incidence rate was observed in the older age group (Fig. 3d).

## Discussion

We summarize the age-adjusted incidence rates and age-specific incidence rates of head and neck cancer, including oral, oropharyngeal, hypopharyngeal, and laryngeal cancer, by time period and birth cohort between 1980 and 2019. The results reflect the effects of the health promotion interventions of the Taiwanese government on cigarette smoking, betel quid chewing, and oral cancer to control head and neck cancer in men. Obvious period and birth effects are observed in oral, oropharyngeal, and hypopharyngeal cancers; however, the most prominent period effect observed between 1990 and 2009 mainly reflects the consumption of betel nuts per capita, while the flattening of the age-adjusted incidence rate of oral, oropharyngeal, and hypopharyngeal cancer since 2010 probably resulted from the declining cigarette smoking rate.

Alcohol consumption, betel quid chewing, and cigarette smoking are three major risk factors for head and neck cancer [3–5]. Each of these risk factors results in different subtypes of head and neck cancers at different levels; betel quid chewing chiefly impacts oral and oropharyngeal cancers, cigarette smoking predominantly influences hypopharyngeal and laryngeal cancers, and alcohol consumption mainly presents as a synergistic factor [12, 13]. A previous paper found that the multivariate-adjusted hazard ratios of betel quid chewing and oral cancer, pharyngeal cancer, and laryngeal cancer were 5.77, 3.30, and 0.97, respectively, while the multivariate-adjusted hazard ratios of cigarette smoking and oral cancer, pharyngeal cancer, and laryngeal cancer were 1.86, 1.72, and 3.04, respectively [14].

The decline of the formerly increasing age-adjusted incidence rate of head and neck cancers can be mainly explained by the great decline in the prevalence rate of cigarette smoking. The age-adjusted incidence rate of oral, oropharyngeal, and hypopharyngeal cancer has flattened since 2010, and the age-adjusted incidence rate of laryngeal cancer has remained steady since 2000 (Fig. 1). The cigarette smoking rates in 1971, 1999, and 2020 were 58.48, 47.3, and 23.1% in males, respectively [15–17]. In addition, the prevalence rate of betel quid chewing declined rapidly after 2000, which might play an important role in the future. The prevalence rate of betel quid chewing in 2002 was 16.8% in males, and it declined to 9.3% in 2017 [18].

The obvious period and similar period effects in oral, oropharyngeal, and hypopharyngeal cancers in men (Fig. 2a-c) can be explained by the long-term trend of betel nuts consumption per capita. A similar pattern of the period effect in the 45- to 64-year age group begins during the 1990–1995 to 2005–2009 time period and then shows a more significant period effect in the 1990–2009 period; however, the period effect lessens after 2010 for oral cancer and hypopharyngeal cancers, while oropharyngeal cancers remain an obvious period effect. According to the statistics analyzed by the Council of Agriculture, betel nuts consumption slightly increases from 0.7 kg per capita in 1971 to 1.4 kg per capita in 1981 [19]. Then, the economy of Taiwan booms significantly, and the gross domestic product increases from 231,397 million dollars in 1970 to 10,328,549 in 2000 [20]. Due to the booming economy, labor-intensity productivity increased, resulting in an increase in betel quid usage. A previous study also showed that less educated older men, blue collar workers, smokers and drinkers have a higher tendency to chew betel nuts [21]. As a result, the consumption of betel nuts per capita significantly increased from 1.4 kg of betel nuts per capita in 1981 to a high of 7.9 kg of betel nuts per capita in 1998 [19]. Then, several anti-betel actions were taken by the government and awareness of the harmful effects of betel quid chewing was more common, and the consumption of betel nuts per capita became steady and started to decline after 2000. Correspondingly, the period effect lessens for oral cancer and hypopharyngeal cancers. Nevertheless, a previous study investigated the oral cancer incidence rates from 1997 to 2016 among men in Taiwan, and suggested that betel nut chewing was the main driver of the cohort effect for oral cancer incidence rates [22]. Even though we obtained the data from the same database, we provided not only a longer time period from 1980 to 2019 but also more categories including oropharyngeal, hypopharyngeal, laryngeal cancer, which result in the different interpretation.

Regarding the maintenance of the obvious period effect of oropharyngeal remains (Fig. 2c), we suspect that HPV has an important effect in the recent time period. In the world, a study from the United States showed that the 50–59 age group had a 2.1% AAPC increase in oropharyngeal SCC among men from 1995 to 2015 [23]. Another study showed a 225% population-level increase in HPV-positive OPSCCs and a 50% decrease in HPV-negative OPSCCs from 1988 to 2004, which also indicates a conservative estimation that 70% of oropharyngeal squamous cell carcinomas will be HPV-positive by 2020 [24]. Another study indicated that HPV-related HNC with an APC of 6.9% rose more rapidly than the incidence of HPV-unrelated HNC with an APC of 5.0%, especially between 40 and 50 years with an APC of 8.5% [25]. Additionally, Wang et al. found that the percentage of HPV positive OPC ranged from 25 to 30% with the lowest between 1999 and 2002 and highest between 2003 and 2010. Focused on male patients, HPV positive OPC has steadily increased from 1.11 to 3.18 per 100,000 person-year for 1999–2002 to 2011–2014. In the same study, HPV subtype varied among 119 HPV positive oropharyngeal cancer with distributions for HPV16, HPV58, HPV35, and HPV33 measuring 70, 12, 3, and 2%, respectively [26].

A clear period effect is shown in the laryngeal cancer of the age period 65–74 years in men (Fig. 2d). There is a slight increase from the time period 1985–1990 to 1990–1994, a significant increase from the time period 1990–1994 to 1995–1999 and a slight decrease from the time period 2000–2004 to 2010–2014. A previous study showed that the multivariate odds ratios were 2.46 and 9.38 for heavy drinking nonsmokers and current smoking nondrinkers, respectively [27]. Hence, the trend can be explained mainly by the declining cigarette smoking rate since 1971 and partially by the rising alcohol drinking rate. The prevalence rates of smoking and alcohol consumption were 31.25 and 41.79% in 1971, 27 and 53.3% in 2002, and 14.5 and 53.4% in 2017, respectively [15, 17, 28].

Due to the high prevalence rate of cigarette smoking and betel quid chewing, the Taiwanese government has put much effort into health promotion, which includes the areca tree replacement program, waste disposal program, and Tobacco Hazards Prevention Act (Table 2). The Tobacco Hazards Prevention Act was implemented in 1997. It not only prohibits teenagers under 18 years old and pregnant women from purchasing cigarettes but also regulates cigarette smoking in the public environment. In addition, tobacco health and welfare surcharges were elevated to 5, 10, and 20 New Taiwan dollar per pack in 2002, 2006, and 2009, respectively; moreover, the tobacco tax also increased to 20 New Taiwan dollar per pack in 2017 [29, 30]. Apart from cigarette smoking, the IARC classified betel quid nut consumption, with or without tobacco, and areca nuts as Class 1 carcinogens in 2004 [31]. Since then, many campaigns have created betel quid-free supportive environments for school campuses, government workplaces, and nongovernmental organizations. The Council of Agriculture of the Taiwanese government then executed the betel nut farming management program to substitute areca trees with other cash crops in 2008. In addition, a waste disposal act that restricted people from spitting betel nut juice in the public environment was implemented in 2017 [32]. Moreover, in addition to the high mortality rate of oral cancer, a population-based screening for oral cancer has been implemented since 2004. People who are both over 30 years old and who chew betel quid or smoke cigarettes or are aboriginal people who are both over 30 years old and who chew betel quid are eligible for free oral mucosal screening every 2 years. As a result, and due to the several interventions mentioned above, the prevalence of cigarette smoking in males decreased from 55.1% in 1996 to 23.1% in 2020, and the prevalence of betel quid chewing in males decreased from 16.76% in 2002 to 9.3% in 2017 [15, 17, 18]. This eventually resulted in a flattened incidence rate of head and neck cancers (Fig. 1). A previous study also showed declining betel quid chewing rates in Taiwan since 2007 and a plateau in the age-standardized incidence rate of oral cancer since 2009 [33]. Another study estimated that the projected incidence rates of oral and oropharyngeal cancer in males will decline after 2025, that the hypopharyngeal cancer rates will plateau after 2030, and that laryngeal cancer in males has decreased since 2004 [8]. Overall, the declining prevalence rate of cigarette smoking since 1996 matches the decline of the formerly increasing age-adjusted incidence rate of head and neck cancers around 2010, which is mentioned above, while the declining betel quid chewing rate is expected to influence head and neck cancer rate in approximately 2025.

**Table 2:** Intervention of Taiwan government to cigarette smoking, betel quid chewing, and oral cancer

Time	Intervention
1997	Tobacco Hazards Prevention Act
2002	Elevates tobacco surcharges to 5 NTD per pack
2004	Free oral cancer screening program
2006	Elevates tobacco surcharges to 10 NTD per pack
2008	Elevates tobacco surcharges to 20 NTD per pack
2009	Betel nut farming management program
2017	Tobacco and Alcohol Tax Act - Elevates tobacco tax to 20 NTD per pack
2017	Waste Disposal Act - Stop people from spitting betelnut juice in public environments

In our study, we analyzed the descriptive epidemiology of head and neck cancer from 1980 to 2019. Similar increasing patterns in the age-adjusted incidence rate, period effect, and cohort effect were observed in oral, oropharyngeal, and hypopharyngeal cancers in men. The strengths of this study are the 40 years of data from a reliable and complete nationwide cancer registry. However, a few limitations should be considered. First, since human papilloma virus is an important risk factor for oropharyngeal cancer, the prevalence rate of human papilloma virus has not been investigated nationally in Taiwan. Second, even though we observed similar birth effects in oral, oropharyngeal, and hypopharyngeal cancers in men, the lack of the prevalence rate by age periods of alcohol, betel quid chewing, and cigarette smoking before 2004 cannot provide enough information for analysis of the effect. Third, birth cohort before 1930–1934 were not analyzed. Choosing class 1930–1934 as our first birth cohort would have the most age periods, and it may also minimize the effect of the lesser life expectancy at birth among the earlier birth cohort while discussing the impact of the major risk factors. Fourth, this study mainly discusses the effect of alcohol consumption, betel quid chewing, and cigarette smoking, and does not address the effect of other risk factors, such as poor oral hygiene, and the impact of lifestyle. Finally, even though the long-term trend of head and neck cancer incidence has massively increased in the past 40 years, the plateau occurred in this decade. Due to the intervention of the government, the strict policies against cigarettes and betel quid indeed flattened the curve of head and neck cancer incidence.

## Conclusions

Rising consumption of betel nuts per capita results in the obvious period effect of oral cavity, oropharyngeal, and hypopharyngeal cancer, while only oropharyngeal cancer maintains the effect due to HPV virus infection. After the strict policy to curb betel quid chewing and cigarette smoking was implemented, not only did the prevalence rate of betel quid chewing and cigarette smoking decline, but the formerly increasing age-adjusted incidence rate of head and neck cancers declined around 2010 for men in Taiwan.

## Data Availability

Publically available Taiwan Cancer Registration data can be accessed at https://twcr.tw/
